# Comparative Study on the Prevention and Control Effects of Rockburst Between Hydraulic Fracturing Sections and Blank Sections

**DOI:** 10.3390/s24227281

**Published:** 2024-11-14

**Authors:** Shuo Yang, Jiang Bian, Aixin Liu, Xiaoyang Li, Fuhong Li, Xingen Ma, Siyuan Gong

**Affiliations:** 1 School of Mines, China University of Mining and Technology, Xuzhou 221116, China; ts21020064a31@cumt.edu.cn (S.Y.); tb24020001a41ky@cumt.edu.cn (J.B.); ts22020037a31@cumt.edu.cn (A.L.); ts23020026a31@cumt.edu.cn (X.L.); 2 State Key Laboratory for Fine Exploration and Intelligent Development of Coal Resources, China University of Mining and Technology, Xuzhou 221116, China; 3 Hetaoyu Coal Mine, Huaneng Qingyang Coal Power Co., Ltd., Qingyang 745300, China; ybklfh@163.com; 4 Huaneng Coal Technology Research Co., Ltd., Beijing 100070, China

**Keywords:** hydraulic fracturing, rockburst, fracturing section, blank section, comparative study

## Abstract

Influenced by various factors such as the complex environment and high key layers in coal mines, hydraulic fracturing technology has gradually become the main means of controlling the hard roof strata to prevent and control rockburst in recent years, which can effectively release the stress on the roof, reduce the intensity of pressure, and ensure the safe and efficient mining of the working face in coal mines. However, the current research on hydraulic fracturing to prevent and control rockburst is mostly limited to optimizing fracturing parameters and monitoring and evaluating fracturing effects, and there are few studies on blank sections, which cannot guarantee the overall prevention and control effect of rockburst, or increase unnecessary construction costs. In this paper, for the directional long borehole staged hydraulic fracturing project, triangular-type blank sections and regular-type blank sections are defined, and the rockburst prevention and control effects of fracturing sections and triangular-type blank sections during fracturing are compared and analyzed by the underground–ground integrated microseismic monitoring technology and transient electromagnetic detection technology, and the rockburst prevention and control effects of fracturing sections and regular-type blank sections during the coal extraction period are compared and analyzed by the underground–ground integrated microseismic monitoring data such as microseismic energy level and frequency as well as the online stress monitoring data. The results show that leaving the triangular-type blank sections can result in reduced construction costs without compromising the effectiveness of rockburst prevention and control. Additionally, the performance of rockburst prevention and control in regular-type blank sections is notably superior to that observed in other working faces without hydraulic fracturing. However, when compared to fracturing sections, the efficacy of rockburst prevention and control in regular-type blank sections remains relatively inferior. Therefore, during the design of fracturing boreholes, it is imperative to strive for maximum coverage of regular-type blank sections. The research findings of this paper comprehensively summarize two prevalent types of blank sections encountered in directional long borehole staged hydraulic fracturing projects. A rigorous comparative analysis is undertaken to evaluate the rockburst prevention and control effects between fractured sections and blank sections. This comparative evaluation serves as a valuable reference for the optimal design of fracturing boreholes, ensuring a balance between achieving effective rockburst prevention and control measures and minimizing economic costs.

## 1. Introduction

In the 1970s, hydraulic fracturing technology began to be applied in oil and gas field development [1,2,3,4,5], which caused the fracture of rock formation through high-pressure injection of fluids and improved the permeability of oil and gas reservoirs and the exploitation efficiency. During the same period, hydraulic fracturing technology was applied in the field of coal mine gas control [6,7,8,9,10], where a large number of fractures formed a fracture network to enhance the permeability of coal seams, improve the effect of underground gas extraction, and reduce the amount of gas outflow from the working face. In the 1990s, hydraulic fracturing technology began to be applied in the field of rockburst prevention and control [11,12,13,14], which reduced the occurrence of rockburst accidents by crushing the hard roof and reducing the degree of stress concentration. Different from the large-diameter roof drilling pressure relief technology [15,16,17] and the roof blasting pressure relief technology [18,19,20], the hard roof hydraulic fracturing technology has lower requirements for the construction environment, has the ability to work on the high roof, and can loosen the coal seam when working on the low roof, effectively improving the coal release rate, and thus has been applied in a large number of coal mines [21,22,23,24,25].

In recent years, many scholars have carried out a lot of research on hydraulic fracturing technology to prevent and control rockburst. In the research of fracturing parameters, Patel et al. [26] estimated the permeability of hydraulic fractures based on the pressure data recorded during hydraulic fracturing, and the estimated results were in general agreement with the results determined by AP608(TM); AlTammar et al. [27] conducted hydraulic fracturing tests on porous specimens under multiple fluid injection sources with an anisotropic stress field, and explored the effect of pore pressure on the expansion of hydraulic fractures; Deb et al. [28] found that rock fracturing can be caused even at low porosity and low permeability by comparing the numerical simulation results of two coupled hydraulic fracturing simulators, CSMP and GEOS; Shang et al. [29] proposed cross-arrangement ground deep and shallow well hydraulic fracturing technology and cross-shot hole ground multi-branch horizontal well hydraulic fracturing technology based on the dynamic disaster mechanism of hard and extra-thick roof induced by hydraulic fracturing; Liu et al. [30] made use of the additional horizontal tensile stress generated by bending deformation of the roof to consider the location of maximum additional horizontal tensile stress as the reasonable fracture location, and the location of the maximum additional horizontal tensile stress was regarded as the reasonable fracture location, and the factors affecting the reasonable fracture location were ranked according to the degree of influence, i.e., roof length > roof elastic modulus > roof thickness > elastic bedding coefficient > horizontal stress; Shi et al. [31] showed that the development of fractures along the direction of the laminar surface was higher than in the direction of the vertical laminar surface, and the fractures were more developed than those in the direction of the vertical laminar surface. The fracture extension is mainly in the form of primary transverse fracture extension, supplemented by longitudinal fracture extension. In terms of the monitoring and evaluation of the fracturing effect, Zhu et al. [32] used microseismic monitoring data to assess the degree of hydraulic fracturing in coal seams by using fine signal processing and interpretation methods, and verified the reliability of the microseismic monitoring method by combining with on-site observation and stress monitoring; Li et al. [33] developed a high-frequency direct current (DC) monitoring system, and the hydraulic fracturing monitoring results showed that the dynamic expansion and spatial shape of hydraulic fractures in rocks can be accurately characterized by the DC monitoring method; Zhong et al. [34] used a fiber-optic grating three-dimensional stress long-term dynamic monitoring system and SOS microseismic system to carry out real-time monitoring of three-dimensional mining-induced stress and microseismic events before and after hydraulic fracturing, and analyzed the changes in the evaluation indexes of the degree of stress concentration in the rock layer of the roof and the large-energy microseismic events after fracturing situations.

Influenced by geological conditions, fracturing process parameters, capital budget, and other factors, some areas of the designed fracturing layer cannot be constructed, resulting in the effect of rockburst prevention in the blank sections being highly dependent on the extension of fractures in the fracturing sections [35,36,37,38]. However, if the fracture extension distance is not enough to cover the blank sections, the stress in the blank sections cannot be adequately released, and there is even the possibility of stress concentration in the blank sections [39,40,41], which increases the risk of rockburst. On the contrary, if part of the area is significantly affected by fracture extension, it should be left as a blank section, and if the hydraulic fracturing operation is carried out, it will increase the construction cost and cause economic loss. However, current scholars mostly focus on the research of fracturing parameters and the monitoring and evaluation of the fracturing effect, and less on the blank section, which leads to the lack of relevant guidance when designing fracturing programs, making it challenging to balance the overall prevention and control effectiveness of rockburst with economic costs.

In order to solve the above problems, this study takes the hydraulic fracturing project of the 1802 working face in Hetaoyu Coal Mine as the research object. The triangular fracturing blind areas formed near curved borehole trajectories are defined as triangular-type blank sections, and the large regular areas not designed for fracturing works are defined as regular-type blank sections. Based on the microseismic monitoring and transient electromagnetic detection data during the fracturing period and the microseismic monitoring and stress monitoring data during the coal mining period, a comparative study was conducted on the rockburst prevention and control effect of the fractured section and the blank section of the 1802 working face. The impact of leaving a blank section on the rockburst prevention and control effect was analyzed, providing a reference for the design of hydraulic fracturing boreholes.

## 2. Project Background and Definition of Blank Sections

### 2.1. Overview of the Mine and 1802 Working Face

Hetaoyu Coal Mine is located in Ningzheng Mining District, Qingyang City, Gansu Province, Northwest China, and belongs to a rockburst mine. Coal dust is explosive, and the coal seams are prone to spontaneous combustion of Class I. Hetaoyu Coal Mine mainly mines eight coal seams; currently, there are two workings faces: 1802 and 2804. The 2804 working face is located in panel I, which has already been mined out and formed a goaf. The 1802 working face is located in panel II, which is in the process of extraction.

The 1802 working face has a strike length of 1900 m, a dip length of 240 m, an average coal thickness of 12.5 m, and an average depth of 974.4 m. The face adopts the method of primary full-height mining, and the roof has collapsed. The roof and floor rocks are both classified as weak to moderately strong, with low rock strength. The maximum horizontal principal stress is greater than the vertical stress, making the floor prone to heaving and the roof susceptible to caving. The eight coal seams where the working face is located have a weak impact tendency, the roof has a weak impact tendency, the floor has no impact tendency, and the impact hazard level during the coal extraction period is medium. The geological structure of the face is complicated, mainly affected by the Beizui-Hetanli downfold, upfold S5, and normal fault F36.

### 2.2. Directional Long Borehole Staged Hydraulic Fracturing

The test ground of the directional long borehole staged hydraulic fracturing project is at the 1802 working face. The fracturing project was carried out at the end mining stage of the 2804 working face, i.e., before the 1802 working face was mined back. The 2804 working face and the 1802 working face were arranged in parallel with each other, and the distance between the coal pillars was far away from each other, which was about 548 m, so the fracturing project was less disturbed by the coal extraction activities of the 2804 working face.

**Figure 1:**
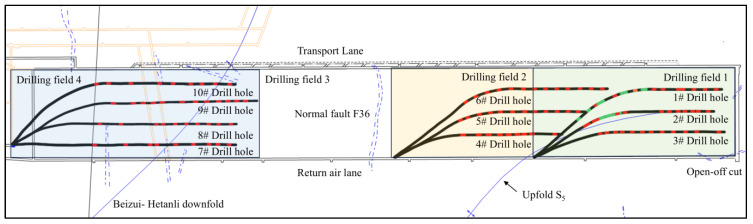
The 1802 workface hydraulic fracturing program.

As shown in Figure 1, the 1802 working face is divided into four areas, or equivalently, four drilling fields are designed, comprising a total of 12 boreholes. This design takes into account the strike length of the 1802 working face, the geological structure of fault F36, the planned drilling depth, and the budget constraints.

Starting from the open-off cut side, the drilling fields are named drilling field 1, drilling field 2, drilling field 3, and drilling field 4. Among them, the hydraulic fracturing project will not be implemented in drilling field 3. Instead, only directional long borehole staged hydraulic fracturing will be conducted in drilling field 1, drilling field 2, and drilling field 4. The boreholes are arranged in a “3-3-0-4” pattern, i.e., three drill holes are arranged in drilling fields 1 and 2, no drill holes are arranged in drilling field 3, and four drill holes are arranged in drilling field 4. The order of borehole fracturing is #3 → #1 → #2 → #4 → #6 → #5 → #8 → #9 → #10 → #7.

Drilling field 1 is located in the initial mining area of the 1802 working face, where the working face passes through the square special structural area and the upfold S5 area, posing a high risk of rockburst. Therefore, three boreholes are arranged in this drilling field. Drilling field 4, on the other hand, is located in the final mining area of the 1802 working face, where the working face passes through the Beizui-Hetanli downfold, encountering numerous small faults and exhibiting a complex geological structure. Moreover, according to the microseismic monitoring data collected during the coal extraction period in the corresponding area of the 2804 working face, this region has experienced multiple high-energy events accompanied by floor heave, indicative of a high risk of rockburst. Therefore, three boreholes are also arranged in this drilling field. In contrast, drilling field 2 and drilling field 3 present relatively lower risks of rockburst. To conduct a comparative analysis of the rockburst prevention effectiveness between the fracturing section and the blank section, three boreholes are arranged in drilling field 2, while no fracturing is performed in drilling field 3.

Generally speaking, the rock layers on the roof of the coal mine are complex, not in the form of layers exposed by the column diagrams of the drill holes [42,43], but in the form of uneven undulations, locally exposed heterogeneous rock bodies, and even the appearance of faults. Therefore, during the fracturing process, the number of designed fracturing sections and the location of fracturing sections were adjusted according to the drilling condition, and the actual fracturing parameters are shown in Table 1.

### 2.3. Definition of Blank Sections

**Figure 2:**
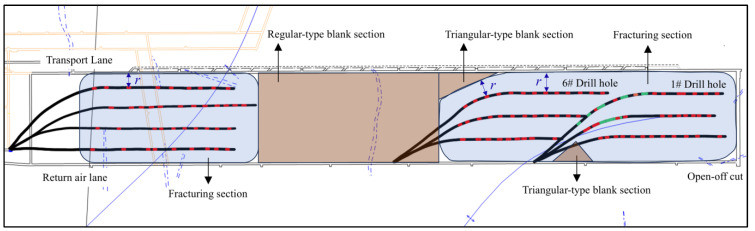
Hydraulic fracturing blank section.

Based on the previously mentioned implementation plan for directional long borehole staged hydraulic fracturing in the 1802 working face of Hetaoyu Coal Mine, this study classifies the blank sections into two types: triangular-type blank sections and regular-type blank sections, as shown in Figure 2.

The directional long hydraulic fracturing project is different from the surface fracturing project in that the construction site can only be selected in the transport lane or return air lane, so some of the borehole trajectories will be curved, which will form a triangular fracture blind zone at the curved area, and the fracture extension radius r cannot be covered, which is defined as a triangular-type blank section in this paper. In Figure 2, a triangular-type blank section on the transport lane side of drilling field 2 is marked. A triangular-type blank section also exists on the transport lane side of drilling field 1, but it disappeared because borehole #6 of drilling field 2 covered the area. The large area created by the absence of a designed borehole in drilling field 3 is defined as a regular-type blank section.

## 3. Comparative Analysis of the Effect of Rockburst Prevention Between Fractured Sections and Triangular-Type Blank Sections During Fracturing

### 3.1. Fracture Monitoring Methods

The directional long borehole staged hydraulic fracturing project in the 1802 working face of Hetaoyu Coal Mine was monitored and evaluated using the underground–ground integrated microseismic monitoring method [44,45] and the transient electromagnetic method [46,47]. During the fracturing process, as the fracture continues to expand, the rock mass around the fractured section is continuously broken, and the accompanying small-energy microseismic events are continuously generated, which are localized by using the underground–ground integrated microseismic monitoring system, and the distribution range of the microseismic events is the fracture extension range. After fracturing, due to the presence of fracturing fluid in the extended fracture, the apparent resistivity of the fracture extension area will be reduced compared with that before fracturing, and the difference between the apparent resistivities before and after fracturing will be detected and calculated by the transient electromagnetic equipment, and a larger difference represents a better fracturing effect. Therefore, the fracturing effect of the fractured sections can be effectively compared with that of the triangular-type blank section by using the underground–ground integrated microseismic monitoring method and the transient electromagnetic method. Since the fracture extension radius obviously cannot cover the regular-type blank section, this paper compares and analyzes the fractured sections with the regular-type blank section based on the multiple types of monitoring data during the coal extraction period in Section 4.

**Figure 3:**
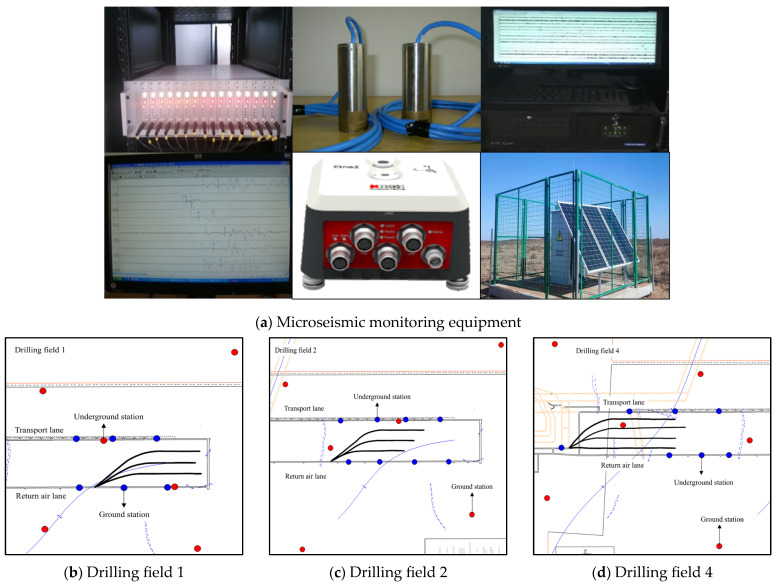
The underground–ground integrated microseismic monitoring system.

Figure 3a shows the wellground integrated microseismic monitoring equipment suite used, with a DLM2001-type pickup seismic sensor downhole and an ET-GSY-type high-precision tridirectional monitor on the ground, which has many advantages such as stability and monitoring accuracy. Figure 3b shows the network layout of the underground–ground integrated microseismic monitoring system in drilling field 1. After the completion of fracturing in the previous drilling field, the ground station and the underground station were moved to the next drilling field so as to obtain the network layout of the underground–ground integrated microseismic monitoring system in drilling field 2, as shown in Figure 3c, and the network layout of the underground–ground integrated microseismic monitoring system in drilling field 4, as shown in Figure 3d. After the hydraulic fracturing project, the ground station and underground station were relocated to drilling field 1, which was used to monitor the microseismic events and evaluate the hydraulic fracturing effect during the coal extraction period.

The transient electromagnetic detection equipment is terraTEM [48]. terraTEM detection equipment has advantages in stability, anti-interference ability, sampling rate, and resolution. It is necessary to judge the fracturing effect based on the difference in apparent resistivity before and after fracturing, so each drilling field needs to be probed before and after fracturing. The presence of fracturing fluid in the fracture after fracturing is the main reason for the decrease in apparent resistivity compared with the pre-fracturing period. Therefore, transient electromagnetic probing should be carried out in a timely manner after the completion of fracturing at the drilling field in order to avoid the loss of fracturing fluid over time or seepage out of the fracture, which will ultimately affect the results of the probing.

**Figure 4:**
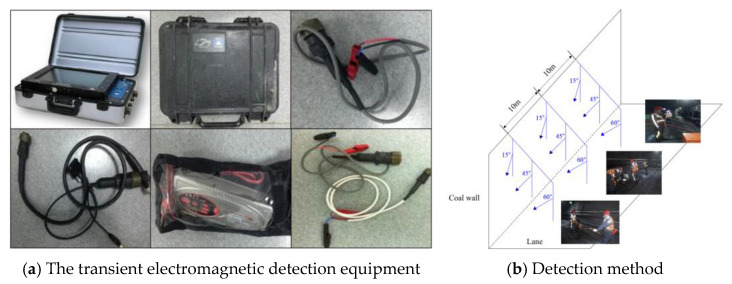
Transient electromagnetic detection.

As shown in Figure 4, transient electromagnetic detection was carried out in both the transport and return air lane, with 10 m intervals between the detection points, and each point was detected at three different angles, i.e., 15°, 45°, and 60° to the coal wall side of the working face, respectively.

### 3.2. Analysis of Fracturing Effectiveness

**Figure 5:**
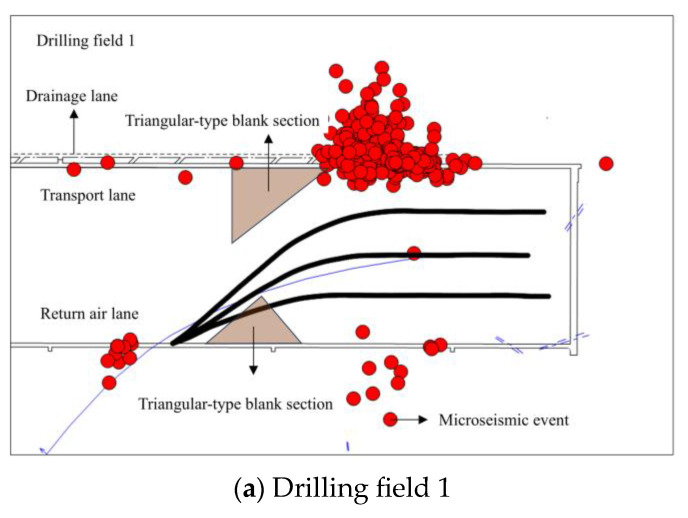

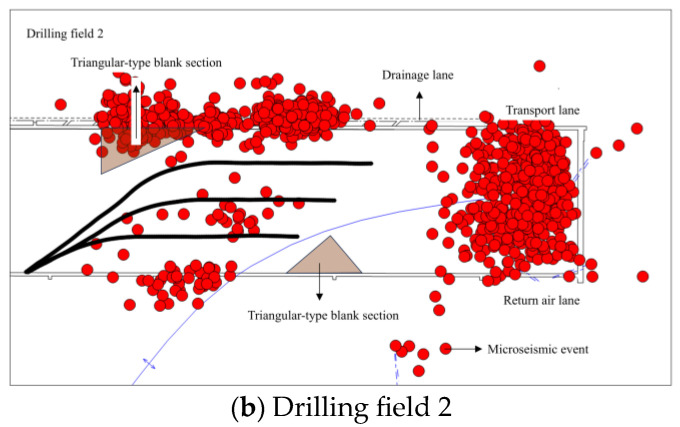
Microseismic distribution during fracturing.

An underground–ground integrated microseismic monitoring system was used to locate the microseismic events generated during the fracturing period, and the distribution of microseismic events between the fractured sections and the triangular-type blank section was plotted according to the locating results; the distribution of microseismic events during the fracturing period of drilling field 1 is shown in Figure 5a, and the distribution of microseismic events during the fracturing period of drilling field 2 is shown in Figure 5b.

**Figure 6:**
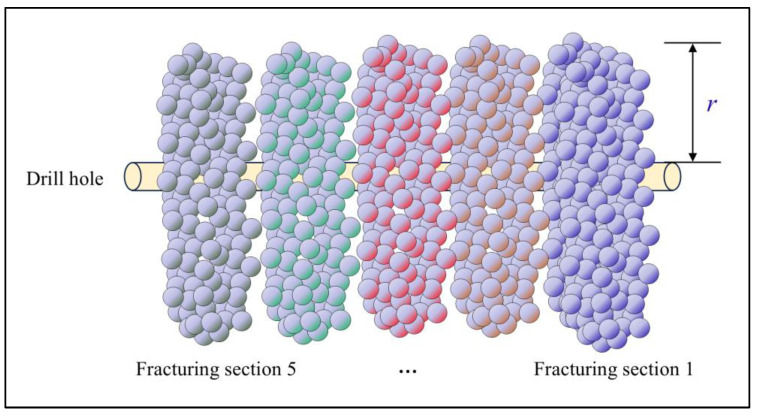
Ideal microseismic monitoring results.

As can be seen from Figure 5, the microseismic events during fracturing in drilling field 1 were concentrated in the vicinity of the transport lane. These microseismic events were primarily generated by the excavation operation of the drainage lane for the transport lane within the 1802 working face, while the remaining events were far-field microseismic events caused by stress perturbations from the hydraulic fracturing operation. The microseismic events during fracturing at drilling field 2 were concentrated near the transport lane and the initial mining area of the 1802 working face, with the former generated by the excavation of the drainage lane and the latter by the extraction operation. The remaining events were far-field microseismic events caused by stress perturbations from the hydraulic fracturing operation. Although Bian et al. [48] solved the theoretical fracture radius of 49.9 m by calculating the Brune model based on the hydraulic fracturing far-field microseismic events, i.e., the fracture extension radius can cover about half of the triangular-type blank sections, this theoretical fracture radius measures the fracture extension of the whole drilling field, and it is not feasible to conduct an accurate assessment specifically targeting the triangular-type fracturing blank sections. Due to the absence of microseismic events generated by direct fracture expansion in the vicinity of the fractured sections, it is not possible to form the ideal monitoring effect shown in Figure 6 for accurately assessing the fracturing effect of the triangular-type blank sections, and therefore, further analyses need to be carried out based on the results of the transient electromagnetic detection.

**Figure 7:**
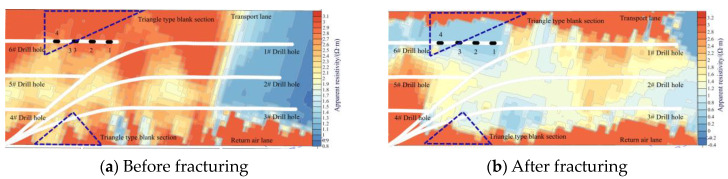
Transient electromagnetic detection results for drilling field 1.

**Figure 8:**
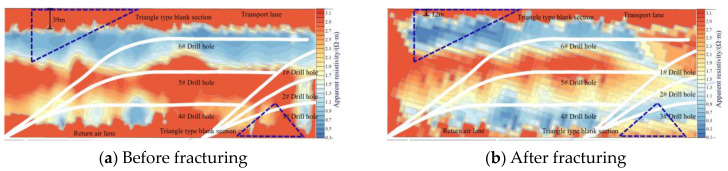
Transient electromagnetic detection results for drilling field 2.

The results of transient electromagnetic detection in drilling field 1 are shown in Figure 7, and the results of transient electromagnetic detection in drilling field 2 are shown in Figure 8. In both figures, the contour fills in the area close to the lanes are in red, which indicates that the resistivity is high, and the resistivity has not changed before and after fracturing because there is more metal equipment, such as mobile brackets, overrunning brackets, and metal pipelines placed in the lanes, which interferes with the results of the detections, and this does not mean that the fracture has not extended to the lanes.

As can be seen from Figure 7, after fracturing in drilling field 1, the triangular-type blank sections are the same as the fractured sections, and the resistivity decreases significantly from 3 Ω-m before fracturing to 1 Ω-m, indicating that the fractures fully expanded in both the fractured sections and the triangular-type blank sections, and that the triangular-type blank sections left behind will not affect the effect of rockburst prevention and control. However, in the directional long borehole staged hydraulic fracturing plan for the 1802 working face of Hetaoyu Coal Mine, borehole #6 was designed to cover the triangular blank section on the transport lane side for hydraulic fracturing. Obviously, after fracturing in drilling field 1, the triangular blank section on the transport lane side has already achieved better fracturing results, and there is no need to repeat fracturing.

As can be seen from Figure 8, before fracturing in drilling field 2, the distance of the low resistance of the triangular-type blank section on the transport lane side from the transport lane was about 39 m, and the distance was reduced to 12 m after fracturing, indicating that after fracturing in drilling field 2, the fracture in the triangular-type blank section on the transport lane side was extended at least to a distance of 12 m from the transport lane, i.e., the fracture has been sufficiently extended both in the fracturing sections and in the triangular-type blank section. The triangular-type blank section on the return air lane side was reduced from 3Ω-m to 1Ω-m before fracturing, which also indicates that the fracture was fully expanded in the triangular-type blank section and the fractured sections, yielding a satisfactory effect.

According to the comprehensive results before and after fracturing in drilling field 1 and drilling field 2, the triangular-type blank sections and the fractured sections are the same. Both have a better fracturing effect, and leaving the triangular-type blank sections without fracturing will not affect the effect of rockburst prevention and control. Therefore, in the directional long borehole staged hydraulic fracturing design plan, the length of the boreholes near the triangular-type blank sections should be appropriately reduced, so as to avoid repeated fracturing within the triangular-type blank sections, which may cause manpower waste and economic loss.

## 4. Analysis of the Effect of Rockburst Prevention and Control in Fractured Sections and Regular-Type Blank Section During the Coal Extraction Period

### 4.1. Analysis of Microseismic Activity Patterns

According to the mining data provided by Hetaoyu Coal Mine, the 1802 working face started to be mined back on 20 September 2023, and has been mined back for 1070.1 m as of 9 June 2024. On 10 March 2024, the working face started to enter the regular-type blank section, and the cumulative footage of the working face at this time was 750.4 m. In addition, the 2804 working face, which was not fractured, started to be mined back in October 2020, and started to be steadily mined back in November 2021, and the cumulative footage of the workface was 749.5 m on 26 September 2022, which is basically the same as the starting position of the regular-type blank section.

Three months of microseismic data from the fractured sections of the 1802 working face, the regular-type blank section of the 1802 working face, and the unfractured area of the 2804 working face were selected for comparative analyses, and a description of the data selection is shown in Table 2.

**Figure 9:**
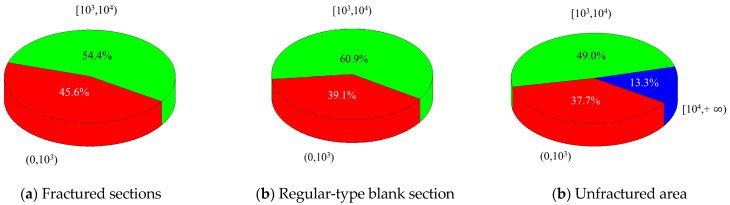
Percentage of microseismic events of different energy levels in different regions.

The results of the collation of microseismic events of different energy levels are shown in Table 3. Compared with the unfractured area of the 2804 working face, the total number of microseismic events, the total energy, and the number of large-energy microseismic events in the fractured sections and the regular-type blank section are significantly reduced, in which the number of high-energy microseismic events of the fourth power and above are all zero, which is more favorable for the prevention and control of rockburst. The total number and energy of microseismic events in the fracturing section are higher than those in the regular-type blank section due to the influence of the excavation operation in the drainage lane of the transport lane. Further comparing the ratio of microseismic events of different energy levels, it can be seen in Figure 9 that the ratio of small-energy microseismic events with energy up to and including the second power is higher than that of the regular-type blank section in the fracturing section, which is characterized by “high-frequency and low-energy”, which is more favorable for the prevention and control of rockburst.

Analyzed in terms of microseismic energy level, the rockburst control effect of the regular-type blank section is significantly better than that of the unfractured area of the 2804 working face, but lower than that of the fractured section.

**Figure 10:**
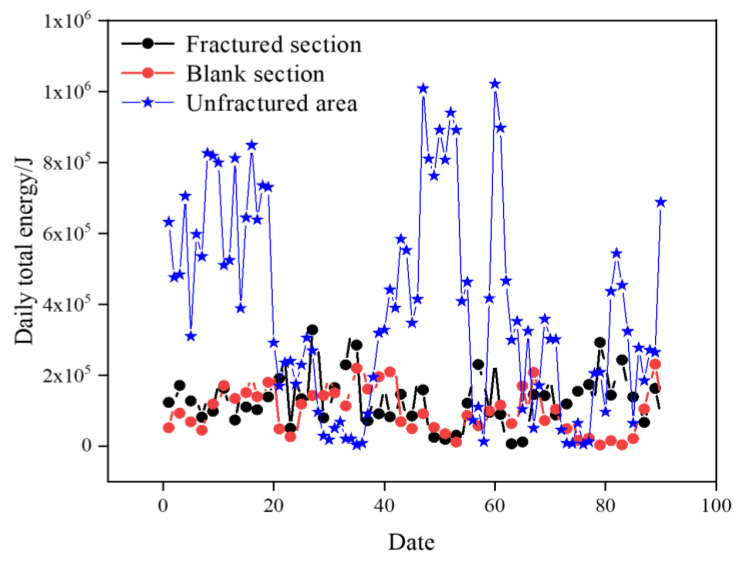
Comparison of daily total energy during the coal extraction period.

A comparison of the daily total energy in different areas is shown in Figure 10. Compared with the unfractured area of the 2804 working face, the daily total energy of microseismicity in the fractured sections and the regular-type blank section is significantly lower, and the daily total energy of the fractured sections and the regular-type blank section is generally lower than 2 × 10^5^ J and not exceeding 4 × 10^5^ J, while that in the unfractured area of the 2804 working face is generally higher than 2 × 10^5^ J and up to about 1 × 10^6^ J, but the contrast between the fractured sections and the regular-type blank section of the 1802 working face is not obvious.

Analyzed in terms of the daily total energy of microseismicity in different areas during the coal extraction period, the effect of rockburst prevention in the regular blank section and the fractured section is significantly better than that in the unfractured area of the 2804 working face.

**Figure 11:**
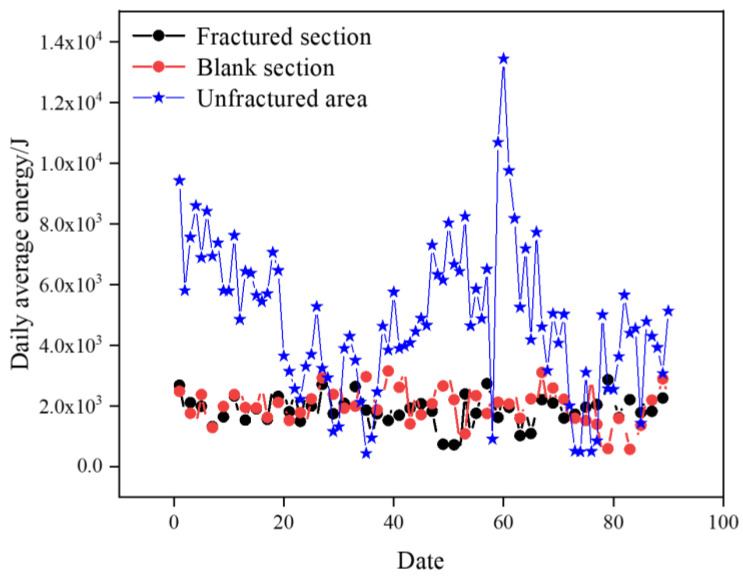
Comparison of daily average energy during the coal extraction period.

A comparison of the daily average energy in different areas is shown in Figure 11. Compared with the unfractured area of the 2804 working face, the daily average energy of microseismic events in the fractured sections and the regular-type blank section is significantly lower, and the daily average energy of the fractured sections and the regular-type blank section is generally around 2 × 10^3^ J, while the daily average energy in the unfractured area of the 2804 working face is generally higher than 4 × 10^3^ J, but the comparison between the fractured sections and the regular-type blank section is not obvious.

From the analysis of the daily average energy of microseismic events in different areas during the coal extraction period, the effect of preventing and controlling rockburst in the regular-type blank section and the fractured sections of the 1802 working face is significantly better than in the unfractured area of the 2804 working face.

**Figure 12:**
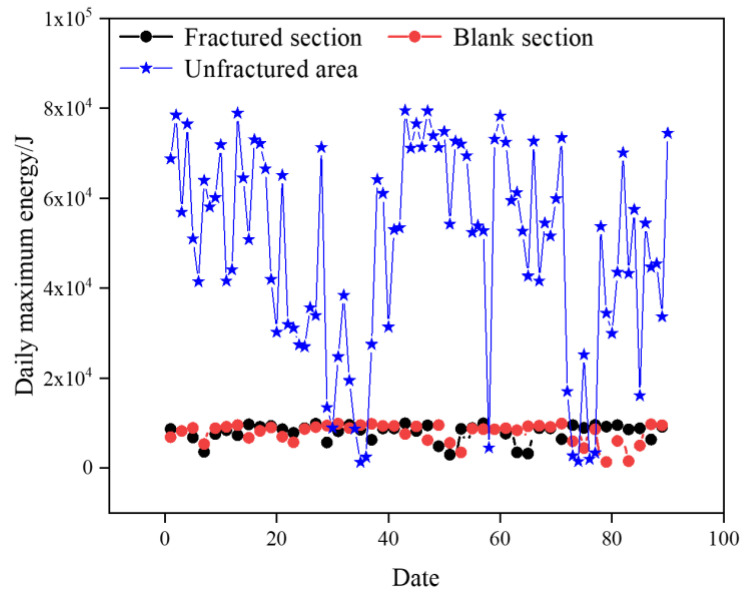
Comparison of daily maximum energy during the coal extraction period.

A comparison of the daily maximum energy in different areas is shown in Figure 12. Compared with the unfractured area in the 2804 working face, the daily maximum energy of microseismic events in the fractured sections and the regular-type blank section is significantly lower, and the daily maximum energy in the fractured sections and the regular-type blank section in the past three months is lower than 1 × 10^4^ J. The daily maximum energy in the unfractured area in the 2804 working face is generally higher than 2 × 10^4^ J, but the comparison between the fractured sections and the regular-type blank section in the 1802 working face is not obvious.

From the analysis of the daily maximum energy of microseismic events in different areas during the coal extraction period, the effect of preventing and controlling the rockburst in the regular-type blank section and the fractured sections is significantly better than that in the unfractured area of the 2804 working face.

**Figure 13:**
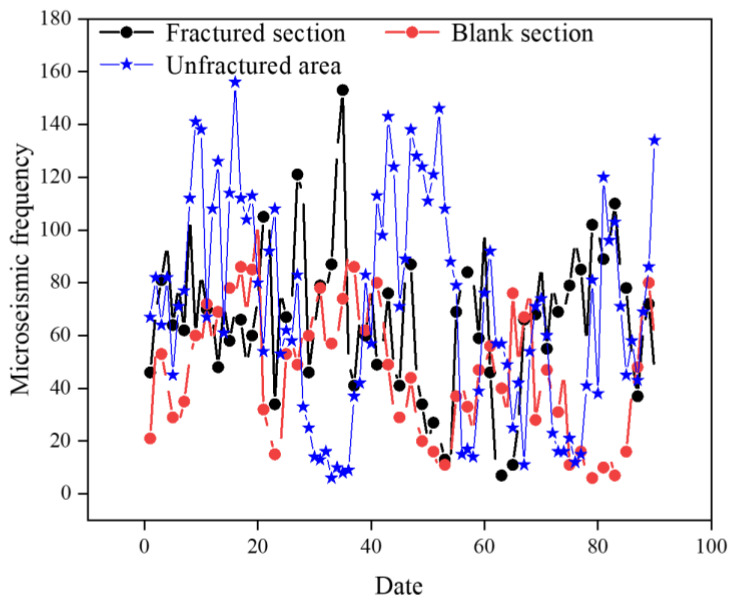
Comparison of microseismic frequency during the coal extraction period.

A comparison of the daily frequency of microseismic events occurring in different areas is shown in Figure 13, revealing that the contrast between the three areas is not pronounced. By calculating the averages, we find that the daily average frequency of microseismic events in the fractured sections is 66.7, the daily average frequency in the regular-type blank section is 45.5, and the daily average frequency in the unfractured area of the 2804 working face is 70.1. In fact, solely based on the fact that the daily average frequency of microseismic events in the fractured section is higher than that in the regular-type blank section, it is not feasible to conclude that the effect of rockburst prevention and control in the fractured sections is superior to that in the regular-type blank section. This is because the fractured sections are also subject to the influence of the excavation operation of the drainage lane, resulting in a higher frequency of microseismic events compared to the regular-type blank section. Furthermore, by considering the total energy of all microseismic events listed in Table 3 and dividing it by the respective number of events, we determine that the average energy of microseismic events in the fractured sections of the 1802 working face is approximately 2.02 × 10^3^ J, the average energy in the regular-type blank section is approximately 2.32 × 10^3^ J, and the average energy in the unfractured area of the 2804 working face is approximately 5.52 × 10^3^ J.

From the analysis of the frequency of microseismic occurrence and the average energy of all microseismic events in different areas during the coal extraction period, the effect of rockburst prevention and control in the regular-type blank section is significantly better than that in the unfractured area of the 2804 working face, but lower than that in the fractured sections.

Based on the comprehensive analysis of microseismic activity patterns such as microseismic energy levels, daily maximum energy, daily average energy, daily total energy, microseismic frequency, and average energy of all microseismic events in different areas, it can be concluded that even if a large area within the working face is reserved without fracturing operations, the rockburst prevention and control effect in this regular-type blank section is significantly better than in other working faces where fracturing engineering has not been implemented, due to the influence of other fractured sections within the working face. However, when compared to the fractured sections, the rockburst prevention and control effect in the regular-type blank section is still relatively low. Therefore, when designing boreholes, it is essential to cover the regular-type blank section as much as possible.

### 4.2. Stress Monitoring Data Analysis

The 1802 working face of Hetaoyu Coal Mine has installed an online stress monitoring system within 300 m ahead of the inner gang of two lanes. Each group of measuring points is arranged with two borehole force gauges and one surrounding rock stress sensor, and the spacing of each group is about 30 m, in which the depth of installation of the shallow base point is 8 m, and the depth of installation of the deep base point is 12 m. The online stress warning indexes of Hetaoyu Coal Mine are as follows: the critical value of the stress at the shallow base point is =12 MPa; the critical value of the stress at the deep base point is =14 MPa.

In the 1802 working face, 6 effective stress-monitoring points were selected to analyze the stress concentration in the fractured sections and the regular-type blank section, of which 118, 119, and 120 measuring points are located in the fractured sections, and 28, 128, and 129 measuring points are located in the regular-type blank section, and the installation location information of each measuring point is shown in Table 4.

As the working face is extracted, the stress change data of each measurement point are as follows.

**Figure 14:**
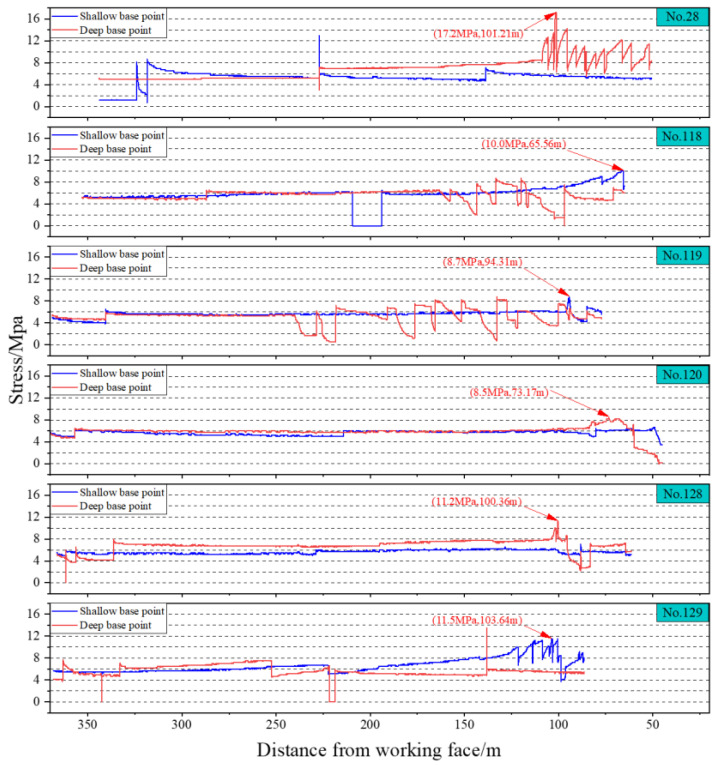
Stress monitoring results.

The ratio of the peak support pressure to the original rock stress is the stress concentration factor [49,50]; the lower the stress concentration factor, the smaller the gathering stress caused by coal extraction, and this is more favorable for the prevention and control of rockburst. The stress concentration factor is calculated from the monitoring results of the stress gauge shown in Figure 14, as shown in Table 5.

Through the analysis of the above data, it can be seen that the stress concentration factor of the fractured sections is within 2, while the stress concentration factor of the regular-type blank section is greater than 2. Therefore, leaving a large area of regular-type blank section is unfavorable for the prevention and control of rockburst, and the subsequent design of boreholes should cover the regular-type blank section as much as possible.

## 5. Discussion and Prospects

### 5.1. Discussion

(1) This paper designs and defines a regular-type blank section for the directional long borehole staged hydraulic fracturing project of the 1802 working face in Hetaoyu Coal Mine, but it should be noted that the premise of designing the regular-type blank section is that the area is relatively safe and the rockburst danger is relatively low. Similarly, if the effect of rockburst pressure prevention and control in the regular-type blank section is more satisfactory, the regular-type blank section should still be selected in a relatively safe area when designing other fracturing programs in the working face.

(2) This paper only compares and analyzes the effect of two types of blank sections on rockburst prevention and control, and does not carry out a more detailed study of the specific details of the area, the number and distribution of blank sections, the impact of the area, and the number and distribution of blank sections on rockburst prevention and control. Such a study can be a better guide to the design of fracturing programs to improve the effectiveness of rockburst prevention and control, and to reduce the economic costs.

(3) There are many methods to evaluate the effect of hydraulic fracturing to prevent rockburst. This paper uses the underground–ground integrated microseismic monitoring and transient electromagnetic detection methods during the fracturing period to analyze the fractured section and the blank section, and uses the microseismic monitoring data and stress monitoring data to analyze the data during the mining period. More detailed investigations can be carried out using other scientific monitoring methods.

### 5.2. Prospects

(1) The hydraulic fracturing project of the 1802 working face studied in this paper is an underground project. Hydraulic fracturing also includes ground hydraulic fracturing and underground–ground integrated hydraulic fracturing, and the research results of this paper can provide a reference for the design of the blank section of the remaining two kinds of fracturing projects.

(2) A comparison of the effects of designing blank sections with different areas, numbers, and distributions on rockburst prevention and control can comprehensively ensure the effectiveness of rockburst prevention and control while further reducing the economic costs, which points out the direction for the next step of in-depth study on the effects of hydraulic fracturing blank sections and fracturing sections on rockburst prevention and control.

(3) With the gradual depletion of coal resources in the shallow central part of the country, it has become an inevitable trend for coal mining to move towards deeper mining, and the accompanying risk of rockburst is also gradually rising, so the means of hydraulic fracturing to prevent and control rockburst will be used more frequently, and the research related to the prevention and control of rockburst in the hydraulic fracturing blank section and the fractured section will be more informative.

## 6. Conclusions

In this study, we take the directional long borehole staged hydraulic fracturing in the 1802 working face of Hetaoyu Coal Mine as the research object and define the triangular-type blank section and regular-type blank section. According to the microseismic monitoring and transient electromagnetic detection data during the fracturing period of the working face and the microseismic monitoring and stress monitoring data during the coal mining period, we further compare and analyze the effect of rockburst prevention and control of the fractured section and the blank section, and obtain the main conclusions as follows:

(1) Comparing and analyzing the effects of rockburst prevention and control between fractured sections and blank sections is a necessary precondition for the design of fracturing boreholes. If the fracture extension distance cannot cover the blank sections, the effectiveness of rockburst prevention and control will be limited, which affects the safety of mining in the working face; if the fracture extension distance can cover the blank sections, but fracturing boreholes have been designed within the area, it will increase unnecessary construction costs, which will result in economic losses.

(2) Restricted by the environmental conditions of the roadway, in the hydraulic fracturing project, there generally exist triangular-type blank sections that cannot be fractured. Comparative analyses of the effect of rockburst prevention and control of the fractured sections and the triangular-type blank sections during the fracturing period were carried out using the underground–ground integrated microseismic monitoring method and the transient electromagnetic detection method. The results show that leaving the triangular-type blank section will not affect the effect of rockburst prevention and control, and the design of fracturing boreholes in triangular-type blank sections should be avoided in hydraulic fracturing projects so as not to increase the construction cost and cause economic loss.

(3) According to the microseismic monitoring data, such as microseismic energy level, microseismic frequency, daily maximum energy, and online stress data during the coal extraction period, a comparative analysis of the rockburst prevention and control effect of fractured sections and regular-type blank section was carried out, and the results showed that although the rockburst prevention and control effect of the regular-type blank section was significantly better than that of the other workings that were not subjected to hydraulic fracturing, the effect of rockburst prevention and control effect was still relatively poor compared with that of the fractured sections. Therefore, in the hydraulic fracturing project, if the financial conditions, construction environment, and other influencing factors permit, the designed boreholes should cover the regular-type blank section as much as possible.

## Figures and Tables

**Table 1:** Fracturing parameters by borehole.

Hole Number	Hole Length/m	Number of Fracturing Sections	Volume of Fracturing Fluid/m^3^	Peak Pressure Range/Mpa	Maximum Pressure Drop Range/Mpa
1	531	12	458.90	22.40–34.90	4.90–12.80
2	522	14	660.05	12.40–33.60	0.70–16.00
3	573	15	581.75	15.20–29.80	2.20–12.90
4	467	9	499.06	19.80–30.30	2.40–8.40
5	546	12	507.94	27.80–37.60	0.90–10.00
6	638	13	694.95	25.50–37.90	2.10–10.60
7	602	9	507.31	10.90–32.00	1.80–19.20
8	620	10	534.93	20.70–33.30	2.70–11.00
9	692	12	614.46	20.10–32.80	1.20–18.20
10	665	9	578.45	10.20–30.80	1.40–16.10

**Table 2:** Microseismic data selection.

Position	Time	Length of Time Window	Distance from the Starting Position to the Open-Off Cut/m
Fractured sections	10 December 2023– 9 March 2024	Three months	332.4
Regular-type blank section	10 March 2024– 9 June 2024	Three months	750.4
Unfractured area of the 2804 working face	26 September 2022–25 December 2022	Three months	749.5

**Table 3:** Microseismic data collation results.

Position	Number of Microseisms of Different Energies			Total Number	Total Energy
	(0, 10^3^)	[10^3^, 10^4^)	$[10^{4},+\infty$)		
Fractured sections	2762	3291	0	6053	1.21 × 10^7^
Regular-type blank section	1667	2595	0	4262	9.49 × 10^6^
Unfractured area of the 2804 working face	2414	3136	851	6401	3.48 × 10^7^

**Table 4:** Borehole stress sensor arrangement.

Sensor Number	Mounting Area	Mounting Depth (i)	Initial Value (i)	Mounting Depth (ii)	Initial Value (ii)
28	Regular-type blank section	8.0	1.2	12.0	5.2
118	Fractured sections	8.0	5.2	12.0	5.2
119	Fractured sections	8.0	5.0	12.0	5.4
120	Fractured sections	8.0	6.0	12.0	5.8
128	Regular-type blank section	8.0	5.5	12.0	5.2
129	Regular-type blank section	8.0	5.7	12.0	4.2

**Table 5:** Borehole stress analysis.

Sensor Number	Mounting Area	Initial Value/Mpa	Peak Support Stress/Mpa	Distance of Peak Point from Working Surface/m	Stress Concentration Factor
28	Regular-type blank section	8.0	1.2	12.0	5.2
118	Fractured sections	8.0	5.2	12.0	5.2
119	Fractured sections	8.0	5.0	12.0	5.4
120	Fractured sections	8.0	6.0	12.0	5.8
128	Regular-type blank section	8.0	5.5	12.0	5.2
129	Regular-type blank section	8.0	5.7	12.0	4.2

## Data Availability

The original contributions presented in this study are included in the article. Further inquiries can be directed to the corresponding authors.
